# Pharyngeal adaptation to bolus properties in patients with Parkinson’s disease

**DOI:** 10.1007/s00405-024-08774-y

**Published:** 2024-06-12

**Authors:** Shakeela Saleem, Anna Miles, Jacqueline Allen

**Affiliations:** 1 Speech Science, School of Psychology, The University of Auckland, B072, Level 2, Building 507, Grafton Campus, Park Road, Private Bag 92019, Auckland, New Zealand; 2 Department of Surgery, The University of Auckland, Auckland, New Zealand

**Keywords:** Parkinson’s disease, Dysphagia, Manometry, High resolution, Modulatory effects

## Abstract

**Purpose:**

Dysphagia is common in people with Parkinson’s disease (PD). Yet, literature describing swallow function in PD using high-resolution manometry is limited. This study explored swallowing pressure metrics for varied bolus conditions in people with PD.

**Method:**

A solid-state unidirectional catheter was used to acquire manometric data for triplicate swallows (5 ml, 10 ml, 20 ml; IDDSI 0, 2 & 4). Penetration-aspiration severity was rated during videofluoroscopy. Patient-reported measures included PDQ-8: Parkinson’s Disease Questionnaire-8 and EAT-10: Eating Assessment Tool-10. Quantitative manometric swallow analysis was completed through Swallow Gateway™. Metrics were compared to published normative values and generalized linear model tests explored modulatory effects.

**Results:**

21 participants (76% male; mean age 69.6 years, SD 7.1) with mild-moderate severity PD were studied. Two patients (9%) aspirated for single bolus thin liquid and paste trials and 15 patients (73%) scored > 3 EAT-10. Standardized PDQ-8 scores correlated with EAT-10 (p < 0.05). Abnormality in UES relaxation and distension was demonstrated by high UES integrated relaxation pressure and low UES maximum admittance (UES MaxAdm) values across varied bolus conditions. Participants demonstrated abnormally elevated pharyngeal contractility and increased post-swallow upper-esophageal sphincter (UES) contractility for thinner liquid trials. Alterations in volume and viscosity had significant effects on the bolus timing metric—distention to contraction latency. UES peak pressure measures were altered in relation to bolus viscosity.

**Conclusion:**

This study identifies early pharyngoesophageal contractile changes in relation to bolus volume and viscosity in PD patients, associated with subtle deterioration of self-reported swallow scores. Manometric evaluation may offer insight into PD-related swallowing changes and help optimize diagnostics and treatment planning

**Supplementary Information:**

The online version contains supplementary material available at 10.1007/s00405-024-08774-y.

## Introduction

Parkinson’s disease (PD) is a progressive neurodegenerative condition, increasing in prevalence [7]. Oropharyngeal dysphagia and weak cough production are common clinical features in individuals with PD, and aspiration pneumonia is reported to be the leading cause of death in PD [3, 10]. The neurophysiological mechanism underpinning swallow dysfunction is complex and often multifactorial. Many studies have described features of oropharyngeal dysfunction in PD using videofluoroscopic swallow studies and/ or flexible endoscopic evaluation of swallowing (FEES). Tongue pumping and tremor, prolonged bolus transit time, delayed swallow initiation, premature spillage, decreased pharyngeal contraction, reduced hyolaryngeal elevation, uncoordinated airway closure during swallowing, diminished or absent cough response to aspiration, reduced laryngeal sensitivity and post-swallow residue in valleculae and pyriform sinus are described clinical features in PD [1, 5, 17].

In recent years, quantitative changes in swallow pressures have been studied by high-resolution manometry (HRM) [4] which measures important aspects of pharyngeal contractility, upper esophageal sphincter (UES) relaxation, and UES opening. However, literature describing swallow function in PD patients using HRIM is limited. Identifying and describing the pathophysiological swallowing changes in PD will support finding specific interventions that best suit to improve function. Modification of bolus consistencies and volume regulation are commonly used compensatory strategies recommended and employed in dysphagia management, aiming to optimize bolus control and coordination, to facilitate safe swallowing. This study aimed to identify changes in pharyngoesophageal swallowing pressure metrics, in patients with PD, as bolus volume and viscosity were altered. In addition we sought to examine these neuromuscular responses in relation to swallow safety and efficiency using the novel core metrics proposed by an international expert group [15].

## Methods

This prospective study was approved by the Health and Disability Ethics Review Committee (HDEC: 19/CEN/131) and written informed consent was obtained from all the participants.

### Study population

Consecutive patients with PD with or without complaint of oropharyngeal dysphagia who were referred to our clinic from November 2019 to May 2022 were invited to participate in this study. All the participants were diagnosed with PD by their neurologists and treated with anti-parkinsonism drugs at recruitment. Parkinson’s symptom severity was rated by Parkinson’s Disease Questionnaire-8 (PDQ-8). PDQ-8 is a shortened version of the original PDQ-39. It contains eight questions providing an overall index of self-perceived health in PD [9]. The questions address the following dimensions: mobility, activities of daily living, emotional well-being, social support, cognitions, communication, bodily discomfort, and stigma. Responses are on a 5-point rating scale from ‘never’ to ‘always’ (scores range from 0 to 40 but are then converted to a score out of 100, with 100 representing the greatest severity). No patients had other diagnosed neurological conditions at the time of recruitment. Participants were excluded if they had swallowing complaint related to other diagnosis (e.g. polymyositis, dermatomyositis and muscle dystrophies) or current/ previous head and neck diagnosis and/or treatment (e.g.: head and neck cancer, cervical osteophytes, enlarged tonsils, external compression such as goiter) or were unable/ unwilling to provide informed consent. Participants were all in an ‘on’ state (taking medications) during assessments.

### Swallow assessment

Within 24 h of the VFSS and EAT-10 [2], participants also completed a HRIM. Participants were fasting for a minimum period of three hours before the examination and were tested while seated in a head-neutral, upright position for pharyngeal swallow evaluations. A 10-French solid-state unidirectional high-resolution manometry catheter (36 pressure sensors spaced at one cm intervals and 16 adjoining impedance sensors each 2 cm) (Model K103659-E-1180-D, Unisensor AG, Attikon, Switzerland) was inserted transnasally with topical anesthesia (cophenylcaine 4%). Once the catheter was positioned correctly (by viewing manometry readings), participants rested for five minutes and an initial accommodation period was observed. A Standardized Bolus Medium (SBM) kit (Trisco Foods Pty Ltd, Brisbane, Australia) which is made in accordance with the International Dysphagia Diet Standardization Initiative (IDDSI) framework (http://iddsi.org/framework/), was used to ensure standardized bolus viscosity and conductivity across different consistencies. SBMkit consists of apple flavoured sodium-chloride concentrate solution, a separate gum-based thickener (Precise Thick’N Instant), and an instruction sheet to make the appropriate IDDSI viscosity levels. A standardized HRIM protocol [15] was followed, with each participant performing triplicate cued swallow trials of nine bolus conditions (total 27 swallows) (thin liquid IDDSI 0, mildly thick liquid IDDSI 2, extremely thick liquid IDDI 4 with three volumes: 5 ml, 10 ml, 20 ml for each consistency). Each bolus was measured and administered to participants via a 20 ml syringe. Before any bolus administration, participants were informed to attempt single swallows per bolus where possible. There was a minimum 20 s break between swallows. On average, an HRIM session lasted 30–40 min.

All HRIM studies were performed by experienced clinicians who had certified training in HRIM, at the swallow research laboratory. The clinician labelled swallows during the procedure for later analysis. Any swallow variations (cough), adverse events, and the protocol completion rate were recorded. In compliance with ethical requirements, each participant was observed for 20 min after completion of the protocol. Once the protocol was completed, raw data was acquired at 20Hz (Solar GI acquisition system, MMS, The Netherlands). All the VFSS data collected based on a standard protocol [11] were rated for penetration-aspiration scale (PAS) [17] score by two expert clinicians.

### Swallow gateway analysis

Pressure and impedance data were exported (ASCII format) and up-loaded (de‐identified) to the online Swallow Gateway™ platform (Flinders Partners Pty Ltd, Australia) for semiautomated analysis. Each participant’s pharyngeal swallows were analysed by a speech-language therapist who had completed the SwallowGateway analysis course. Uploaded data were analyzed using varied landmarks and validated metrics as described in Table 1 [4, 8, 16]. Analytic methods and reliability of SwallowGateway analysis have previously been described [4]. Some participants’ swallow data with recording errors (new equipment technical errors, swallow labelling error) were excluded from the final analysis. 50% of randomly selected HRIM data were analyzed by a second rater. Both raters were blinded to clinical characteristics. Markers placements were thoroughly reviewed by both the raters and difficult swallow analysis were resolved through consensus and referring to the expert tips.

**Table 1:** HRIM pressure flow parameters and definitions for core outcome metrics

Measures	Abbreviation	Description (unit) [8, 15]
Pharyngeal contractile integral	PHCI	An integral pressure measure of pharyngeal contractile vigour spanning from the velopharynx to the upper margin of the UES (in mmHg.cm.s)
Velopharyngeal contractile integral	VCI	An integral pressure measure of pharyngeal contractile vigour spanning the velopharyngeal region only (in mmHg.cm.s)
Mesopharyngeal contractile integral	MCI	An integral pressure measure of pharyngeal contractile vigour spanning the mesopharyngeal region only (in mmHg.cm.s)
Hypopharyngeal contractile integral	HPCI	An integral pressure measure of pharyngeal contractile vigour spanning the hypopharyngeal region only (in mmHg.cm.s)
Intra-bolus distension pressure	IBP	The pressure 1 cm superior to the UES apogee position at the time of maximum hypopharyngeal distension (indicated by impedance/admittance) (in mmHg)
UES integrated relaxation pressure	UES IRP	A pressure measure of the extent of UES relaxation pressure, generated as the median of the lowest pressure in a non-consecutive 0.20–0.25 s window (in mmHg)
UES relaxation time	UES RT	A measure of the duration of UES relaxation – a pressure interval below 50% of baseline or 35 mmHg, whichever is lower, in units of seconds (in s)
UES maximum admittance	UES MaxAdm	A measure of extent of UES opening. The highest admittance value (inverse of impedance) recorded during trans-sphincteric bolus flow (in millisiemens-mS)
Upper oesophageal sphincter contractile integral	UES CI	An integral pressure measure of UES contractile vigour, post swallow (in mmHg.cm.s)
UES basal pressure	UES BP	The peak pressure at the level of the UES pre-swallow (in mmHg)
UES peak pressure	UES PeakP	The peak pressure at the level of the UES measured immediately post pharyngeal contraction (in mmHg)
Pharyngeal Distension-Contraction Latency	DCL	A timing measure from maximum pharyngeal distension to the pharyngeal luminal occlusive contraction—a correlate of how well the bolus is propelled ahead of the pharyngeal stripping wave (in mS)
Bolus Presence Time	BPT	The dwell time of the bolus in the pharynx (in mS)
Swallow Risk Index	SRI	A composite formula score designed to capitalise on the directionality of aberrant swallow parameters. The original report described SRI in patients with neuromuscular disease and aspiration on radiology

### Statistical analysis

The average results for the triplicate swallows for nine bolus conditions (3 volumes and 3 consistencies) were tabulated in an Excel spreadsheet for statistical analysis using SPSS (IBM Corp., IBM Statistical Package for the Social Sciences (SPSS), v 27.0 Armonk, NY, IBM Corp). Individual values for each parameter was compared to Swallowgateway normative study data (www.swallowgateway.com) [14] and counted for abnormality if it was below 5th and above 95th percentile. As our study didn’t have a control group to compare the findings we selected swallowgateway norms as the values were derived from similar methods and equipment configuration to our study (same catheter, similar protocol and same bolus medium used for the oral trials). Association between disease severity and bolus characteristics was assessed using Spearman rank order correlation. Volume and viscosity main effects were evaluated using general linear mixed model repeated measures analysis. Bonferroni adjustment was applied to pairwise comparisons. A *p*-value of < 0.05 was considered to indicate statistical significance.

## Results

All HRIM procedures were completed without complications. Twenty-one participants (5 females) completed HRIM with a mean age of 69 years (SD 7 years). The patient characteristics are shown in Table 2. Participants all demonstrated mild PD severity by the Hoehn and Yahr rating and PDQ-8 scores. The manometry procedure was well-tolerated by all the participants and no adverse effects were recorded across the cohort.

**Table 2:** Participant demographics characteristics and self-reported disease severity scores

Variables	Mean ± SD (Range)
Age group	69.67 ± 7.16 (59–86)
Sex (male: female)	16:5
Years of diagnosis	7.67 ± 6.39 (1–18)
Parkinson’s Disease Questionnaire-8 (Standardized score)	29.18 ± 15.92 (0–50)
Eating Assessment Tool-10 score	7.76 ± 6.75 (0–26)

PDQ-8 severity—0 = no impact of symptoms, 100 = maximum impact association; Hoehn and Yahr (1967) staging—H&Y I (17.74), H&Y II (33.14), H&Y III (37.05), H&Y IV (47.86)

### Dysphagia severity

**Fig. 1:**
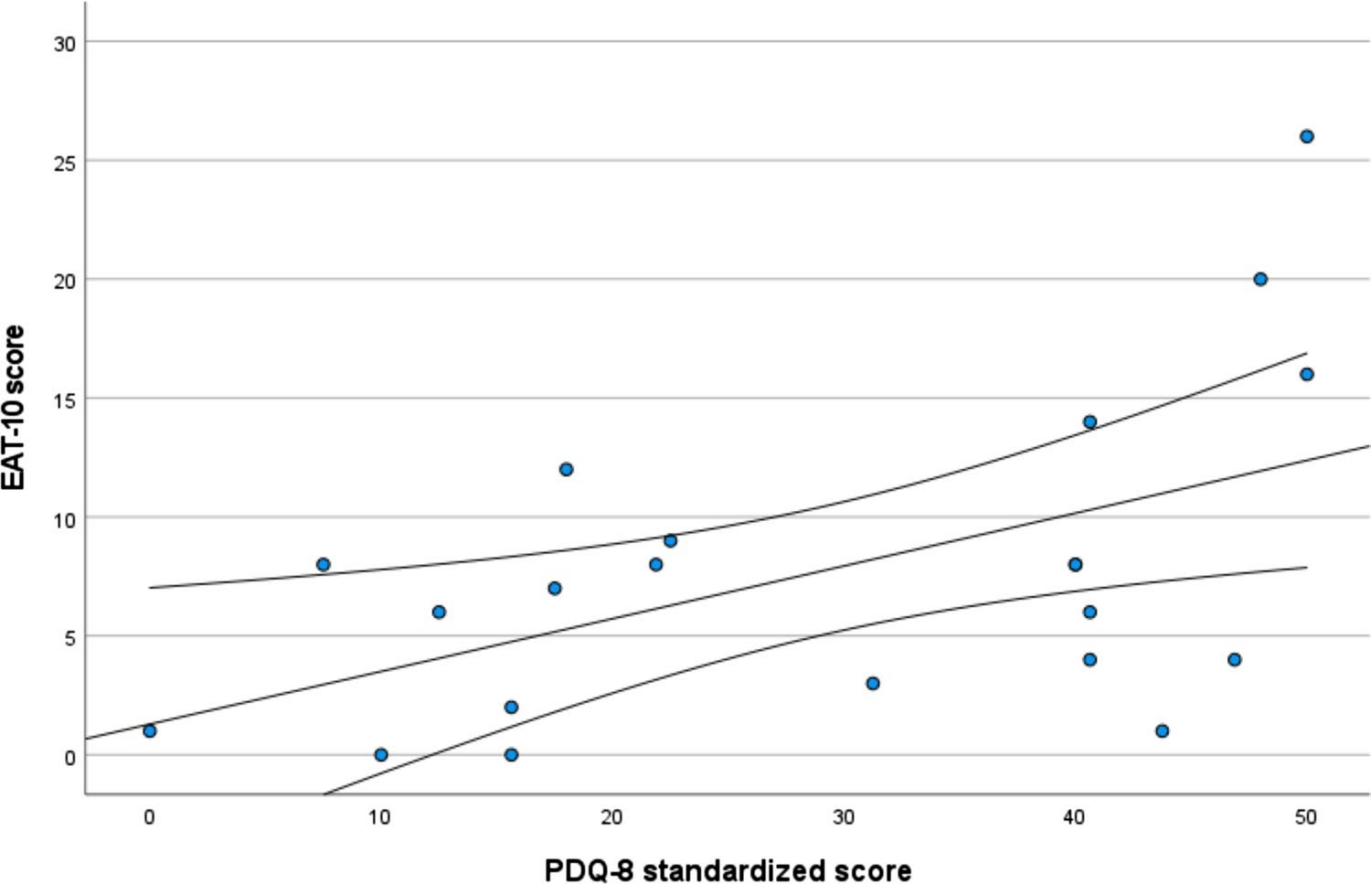
Positive correlation between Parkinson’s severity (PDQ-8) and dysphagia severity (EAT-10). Individuals score with a fitted regression line with 95% confidence interval

All participants reported eating a normal diet with no modifications to food and drinks (IDDSI Level 7). However, 73% of participants (n = 15/21) reported swallowing disturbances and scored outside the normal range (> 3 points) on the EAT-10. In the VFSS examination, four participants aspirated (PAS 7–8) during the 100 ml straw drinking task (and two of them aspirated for 3 ml (P006), 20 ml (P036) and 3 cm^3^ (P036) paste bolus trials). The remaining seventeen participants had normal PAS scores for all swallow bolus trials. Self-reported PDQ-8 score was positively associated with self-reported dysphagia severity assessed by EAT-10 scores (r_s_ (19) = 0.504, *p* = 0.02) (Fig. 1).

### HRIM parameters

*IDDSI 0 bolus trials*: compared to (below 5th and above 95th percentile) normative measures, participants demonstrated abnormally elevated mesopharyngeal contractile integral (n = 5/21), elevated hypopharyngeal contractile integral (n = 6/21), high UES integrated relaxation pressure (UES IRP) (n = 5/21) and low UES maximum admittance (UES MaxAdm) for 20 ml swallow trials. In other volume trials (5 ml, 10 ml) UES IRP (n = 7/21) and UES contractile integral (n = 5/21) were elevated in roughly a quarter to one third of participants.

*IDDSI 2 and IDDSI 4 bolus trials*: a greater number of participants had abnormally low UES MaxAdm with increased viscosity bolus (IDDSI 2: n = 10/21 and IDDSI 4: 8/21). Hypercontractility in the mesopharynx, hypopharynx, and elevated UES IRP was infrequent (n = 2, n = 3 and n = 4 respectively) for other consistencies. (All abnormal patients scores across volumes and viscosity are presented in Supplementary Table 1, and HRIM metrics means with 95% confidence intervals are presented in Supplementary Table 2.

### Bolus volume and consistency

**Fig. 2:**
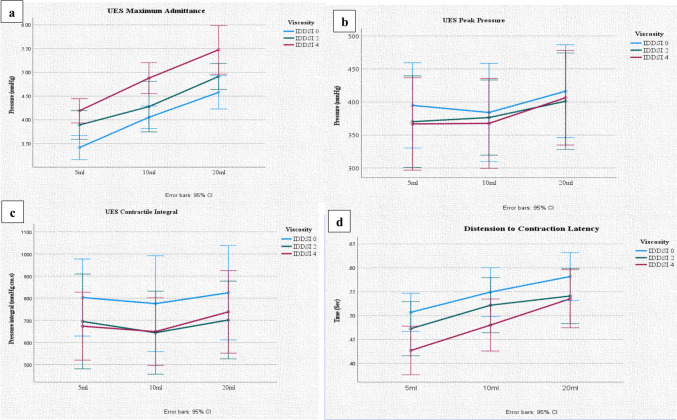
Graphs showing effects of volume and viscosity for **a** UES maximum admittance, **b** Distension to contraction latency, **c** UES Contractile integral, and **d** UES peak pressure

Both volume and viscosity increased UES maximum admittance (respectively F = 67.59, *p* < 0.001; F = 21.39, *p* < 0.001) (Table 3). The timing measure, distension to contraction latency (DCL), increased as the bolus volume increased (F = 31.93, *p* < 0.001). In contrast, bolus viscosity significantly reduced DCL (F = 11.50, *p* < 0.001). UES contractile integral demonstrated a significant correlation with bolus consistency, whereby reduced UES contractility was seen with increased viscosity (F = 6.80, *p* = 0.03). Bolus viscosity reduced the UES peak pressure (F = 5.25, *p* = 0.011). No other HRIM measures differed significantly in relation to increased volume and viscosity. Comparison plots are displayed in Fig. 2 for variables UES MaxAdm, DCL, UES PeakP, and UES CI.

**Table 3:** Summary of the main effects of volume and viscosity

Measures	Volume effect		Viscosity effects	
	*F*	*P*	*F*	*P*
Pharyngeal contractile integral (PHCI)	1.452	0.251	0.305	0.657
Velopharyngeal contractile integral (VCI)	2.280	0.138	2.237	0.145
Mesopharyngeal contractile integral (MCI)	1.421	0.252	0.038	0.934
Hypopharyngeal contractile integral (HPCI)	0.910	0.400	0.368	0.620
Intra-bolus distension pressure (IBP)	1.280	0.273	0.916	0.352
UES integrated relaxation pressure (UES IRP)	1.607	0.219	1.075	0.344
UES relaxation time (UES RT)	1.215	0.284	0.973	0.336
UES maximum admittance (UES MaxAdm)	**↑ 67.596**	**< 0.001**	**↑ 21.394**	**< 0.001**
UES contractile integral (UES CI)	2.350	0.116	**↓ 6.802**	**0.003**
UES basal pressure (UES BP)	1.782	0.188	2.234	0.133
UES peak pressure (UES PeakP)	0.699	0.459	**↓ 5.253**	**0.011**
Pharyngeal Distension-Contraction Latency (DCL)	**↑ 31.932**	**< 0.001**	**↓ 11.504**	**< 0.001**
Bolus Presence Time (BPT)	0.995	0.330	1.053	0.317
Swallow Risk Index (SRI)	0.981	0.335	0.941	0.345

F statistics and P values of general linear model repeated measures analysis are presented. ↑ Indicates directionality of effects on volume and viscosity. Bold results indicate metrics that showed statistical significance following the Bonferroni adjustment. UES: Upper esophageal sphincter

No relationship between PDQ-8 severity and any of the HRIM measures was found. EAT-10 score was correlated with the following measures: UES basal pressure was significantly higher for (*p* = 0.04) thin liquid bolus trials in the group with elevated EAT-10 scores (≥ 7), velopharyngeal contractile integral was significantly lower (*p* = 0.04) for extremely thick liquid trials (IDDSI 4) in individuals with elevated EAT-10 scores and UES MaxAdm was significantly lower (*p* < 0.05) across most bolus conditions in the group with elevated EAT-10 scores (Supplementary Table 3).

## Discussion

The present study explored pharyngoesophageal swallowing function in individuals with PD using HRIM metrics and characterized pharyngeal modulation of swallow parameters in relation to a wide range of bolus conditions. Key findings of the study were that individuals with PD presented with increased pharyngeal contractility (MCI, HPCI), abnormality in UES relaxation and distension (high UES IRP, low UES MaxAdm), and increased post-swallow UES contractility (UES CI). Both volume and viscosity changes had significant modulatory effects on UES maximum admittance and distension to contraction latency metrics, and UES CI and UES peak pressure measures were altered in relation to bolus viscosity.

In this current cohort of individuals with PD, self-reported dysphagia symptoms were prevalent in 73% of individuals but participants maintained unrestricted diets. Only four (19%) demonstrated abnormal penetration-aspiration scores during VFSS examination. No participant was being treated by a speech-language therapist for swallow dysfunction at the point of recruitment. This suggests that early swallow changes occur and are identifiable prior to individuals reporting or seeking dysphagia-related professional support. There was a significant positive correlation between self-reported Parkinson’s disease severity (PDQ-8) and self-reported dysphagia severity (Fig. 1). This finding is supported by other studies reporting advancing swallow impairment as PD stage advances [12, 19, 20].

Increased pharyngeal contractility in the mesopharyngeal and hypopharyngeal regions were exhibited in this cohort of PD patients, a finding mirrored by Szczesniak et al. in their recent study of 64 PD patients [21]. This may indicate an adaptive behaviour by the pharynx to achieve effective bolus transfer and prevent pharyngeal residue accumulation, given that we also found reduction in UES MaxAdm which represents restriction to flow across the UES. A recent study in *normal* adults, using a similar HRIM protocol to our own, by Ferris et al. [8] also demonstrated velopharyngeal, hypopharyngeal, and pharyngeal contractility increased during larger bolus volume trials and with increased bolus viscosity, suggesting that more intense force is generated in the pharyngeal chamber for propulsion of larger bolus volumes. Reduced force generation in the face of larger bolus volumes or increased bolus viscosity, due to weak pharyngeal muscles or muscle atrophy in the PD population, potentially results in inefficient pharyngeal constriction and less bolus impetus, slower bolus velocity and risks residue being left behind post-swallow. Several previous videofluoroscopic studies report reduced pharyngeal constriction as one of the key features associated with compromised swallow efficiency (residue) in patients with PD [1, 6, 19].

Ferris also reported adaptation of hypopharyngeal intrabolus distension pressure (IBP) for varied bolus conditions in neurologically healthy adults [8] but we did not identify this in our PD group. This may imply pharyngeal weakness in our cohort or loss of modulation skill or ability, given the lack of increase in pharyngeal contractility detected with presentation of increased volume and viscosity.

Elevated UES integrated relaxation pressure (UES IRP) was observed in our cohort, in keeping with previous study findings [21]. Resistance to bolus flow across the UES is influenced by the adequacy of UES relaxation, extent of UES opening (contingent on hyolaryngeal motion), and duration of UES relaxation [4]. Elevated UES IRP indicates abnormal flow resistance in these PD patients. Healthy adults demonstrated elevated UES IRP in response to increased volume and viscosity [8]. In our patients, although we saw an overall elevated UES IRP, this did not vary with differing bolus volumes or viscosities, but remained elevated throughout (Supplementary Table 1). Explanation for this may include that UES resistance may be a fixed aspect of the muscle or hyolaryngeal system, or represent loss of an adaptive response. If the UES had become fibrotic and non-compliant we would have expected solid bolus to also be restricted in passage. This consistency was not tested during this protocol, and we are unable to ascertain whether solid food transit impairment was present (although few participants complained of this). This may be relevant when considering intervention, as exercise therapy in this setting may need to be specific to hyolaryngeal movement, or may be less effective if a non-compliant muscle is present, in which case direct UES dilatation might offer greater benefit.

Interestingly, we observed that larger bolus volumes and increased bolus viscosity were associated with greater UES maximum admittance (UES MaxAdm) in this cohort, but that overall our participants demonstrated low UESMaxAdm. UES MaxAdm is a marker of the extent of UES opening, and low admittance values indicate reduced excursion of the UES producing increased bolus flow resistance [4]. The UES can also be forced open by hydrostatic pressure force if a large bolus is taken, *if* all other exits from the pharynx are closed (i.e. nasopharynx and airway). In this case, tongue pumping behaviour can drive a large bolus through the UES in a piecemeal fashion. In our study, the increase in UES MaxAdm (although not in the normal range) suggests the UES is still compliant but has not been distracted adequately (due to poor hyolaryngeal function) and the greater bolus volume is providing some hydrostatic force to drive bolus across the PES. There may still be an aspect of reduced compliance of the UES itself as connective tissue in the muscle becomes more rigid with age, and combined with lack of distraction related to reduced hyolaryngeal elevation, results in reduction in overall UES opening extent and duration. In PD, reduced oral-phase pressure generation due to bradykinetic lingual movement, premature spillage due to reduced tongue posterior bolus containment, delayed swallow reflex, increased pharyngeal transit time, and reduced hyolaryngeal excursion may be contributing to poor bolus impetus and reduced UES opening. These findings have been reported in videofluoroscopic swallow studies in PD previously [1, 17]. A combination of factors is likely to emerge in each individual, that dictates overall pharyngeal transit. Effects of bolus volume and viscosity on UES MaxAdm evident in this cohort are consistent with previous work [22] and also with values in neurologically healthy individuals where UES MaxAdm varied across volumes and viscosity [8].

Post-swallow UES contractility was abnormally elevated in a greater number of PD patients for 5ml thin liquid bolus trials, compared to larger volumes and thicker bolus trials. Potential explanations for these differences include an immediate post-swallow contraction to help prevent retrograde flow of thin bolus, which might otherwise easily regurgitate, if muscle weakness is present, or that if the pharynx receives a liquid volume bolus, it requires greater concentric clearing contraction to reach it (as liquid hugs the pharyngeal contours) and provide propulsion compared to a larger, less free-flowing and more cohesive bolus, where pharyngeal wall constriction is able to contact the bolus at an earlier timepoint. These mechanisms may also explain elevated UES peak pressure for thin bolus compared to thick. The lack of change when different volumes were presented is interesting, and may reflect gravity influence on thin liquids wherein the natural velocity of the fluid is greater or just the capacity of the pharynx overall to manage volumes within the 5–25 ml range. The hydrostatic pressure generated by a larger liquid bolus size would also increase liquid velocity through inertia and thus the pharynx may not need to apply additional pressure to the larger liquid bolus. Interestingly, neurologically healthy adults did not alter UES contractility or UES peak pressure in relation to bolus viscosity possibly suggesting redundancy in the ability of the normal pharynx to generate pressures across a range that enables consistent flow rates through the UES [8, 13].

Our study shows that pharyngeal distension to contraction latency (DCL) significantly altered in relation to both bolus volume and viscosity. A longer duration of DCL was seen with increased volumes and a shorter duration of DCL was observed with increased viscosity, in line with previous study findings in normal adults [8, 13]. Greater volume bolus will take longer to distend the pharynx as it fills, increasing the DCL whereas a more viscous but same-sized bolus will require more impetus to travel the pharynx and through the UES in the same timeframe, therefore contraction is occurring earlier to ensure flow rate consistency and bolus transit times remain normal. Taken together, these findings suggest that pharyngeal swallow sensory-motor regulation is still preserved in the early stages of PD.

### Study limitations and future directions

This study described preliminary findings of manometrically characterized pharyngoesophageal swallow changes in a small cohort (n = 21) of early/mid-stage PD patients. Although we aimed to recruit participants with a range of PD severity stages, study data collection was affected by the COVID-19 pandemic. Therefore, our findings cannot be generalized to more severe stages of PD. Future studies should include a larger sample size with a wider range of disease severity. This study did not exclude patients with normal EAT-10 scores. However, future larger group studies should consider including more homogeneous groups either with or without complaint of swallowing difficulties in order to provide stronger analysis. We presented bolus conditions with varied volumes and viscosity in a fixed order rather than providing it in random order. Hence, data may have been influenced by time-order and fatigue effects. We haven’t included an age-matched control group, however, we were able to compared our HRIM measures with normative data provided by the open-source SwallowGateway web-based application which utilizes the same hardware as our laboratory. This application includes data across all ages (≥ 18 years) and may not be specifically generalizable to older people.

## Conclusions

Using HRIM evaluation we were able to describe changes in pharyngeal swallow behaviour and responses triggered by altering bolus volume and viscosity conditions in PD patients. Key swallow modulatory changes in PD patients include greater resistance to flow (decreased UES MaxAdm and elevated UES IRP) with corresponding increased pharyngeal contractility and elevated IBP. Lack of responsiveness of UES IRP and IBP to bolus parameter change, suggests either, that hyolaryngeal movement was impaired and unable to provide normal UES distraction or that fixed resistance may be present at the UES contributing to increased resistance. Implications of these findings when planning management suggest that exercise-based therapy alone may not address early swallow changes and that targeted therapy at the UES may also have a role to play in reducing resistance to bolus flow. Use of HRIM evaluation in PD individuals would help guide the choice of treatments, and may provide further insights into PD-related dysphagia to help optimize the diagnostic and treatment planning framework.

## Supplementary Information

Below is the link to the electronic supplementary material.

Supplementary file1 (DOCX 32 KB)

## References

[1] Allen JE, Miles A (2020) Parkinson disease. In: Weissbrod PA, Francis DO (eds) Neurologic and neurodegenerative diseases of the larynx. Springer International Publishing, Cham, pp 143–159

[2] Belafsky PC, Mouadeb DA, Rees CJ, Pryor JC, Postma GN, Allen J, Leonard RJ (2008) Validity and reliability of the Eating Assessment Tool (EAT-10). Ann Otol Rhinol Laryngol 117(12):919–924. 10.1177/000348940811701210 19140539 10.1177/000348940811701210

[3] Beyer MK, Herlofson K, Arsland D, Larsen JP (2001) Causes of death in a community-based study of Parkinson’s disease. Acta Neurol Scand 103(1):7–11. 10.1034/j.1600-0404.2001.00191.x 11153892 10.1034/j.1600-0404.2001.00191.x

[4] Cock C, Omari T (2017) Diagnosis of swallowing disorders: how we interpret pharyngeal manometry. Curr Gastroenterol Rep 19(3):11. 10.1007/s11894-017-0552-2 28289859 10.1007/s11894-017-0552-2 PMC5348549

[5] Cosentino G, Avenali M, Schindler A, Pizzorni N, Montomoli C, Abbruzzese G et al (2022) A multinational consensus on dysphagia in Parkinson’s disease: screening, diagnosis and prognostic value. J Neurol 269(3):1335–1352. 10.1007/s00415-021-10739-8 34417870 10.1007/s00415-021-10739-8 PMC8857094

[6] Curtis JA, Molfenter SM, Troche MS (2020) Pharyngeal area changes in Parkinson’s disease and its effect on swallowing safety, efficiency, and kinematics. Dysphagia 35(2):389–398. 10.1007/s00455-019-10052-7 31446478 10.1007/s00455-019-10052-7 PMC7513198

[7] Feigin VL, Abajobir AA, Abate KH, Abd-Allah F, Abdulle AM, Abera SF et al (2017) Global, regional, and national burden of neurological disorders during 1990–2015: a systematic analysis for the Global Burden of Disease Study 2015. Lancet Neurol 16(11):877–897. 10.1016/S1474-4422(17)30299-5 28931491 10.1016/S1474-4422(17)30299-5 PMC5641502

[8] Ferris L, Doeltgen S, Cock C, Rommel N, Schar M, Carrión S et al (2021) Modulation of pharyngeal swallowing by bolus volume and viscosity. Am J Physiol Gastrointest Liver Physiol 320(1):G43–G53. 10.1152/ajpgi.00270.2020 33112160 10.1152/ajpgi.00270.2020

[9] Jenkinson C, Fitzpatrick R, Peto V, Greenhall R, Hyman N (1997) The PDQ-8: development and validation of a short-form Parkinson’s disease questionnaire. Psychol Health 12(6):805–814. 10.1080/08870449708406741

[10] Kalf JG, de Swart BJ, Bloem BR, Munneke M (2012) Prevalence of oropharyngeal dysphagia in Parkinson’s disease: a meta-analysis. Parkinsonism Relat Disord 18(4):311–315. 10.1016/j.parkreldis.2011.11.006 22137459 10.1016/j.parkreldis.2011.11.006

[11] Leonard R, Kendall KA (2019) Dysphagia assessment and treatment planning: a team approach, 4th edn. Plural Publishing Inc., San Diego

[12] Miller N, Allcock L, Hildreth AJ, Jones D, Noble E, Burn DJ (2009) Swallowing problems in Parkinson disease: frequency and clinical correlates. J Neurol Neurosurg Psychiatry 80(9):1047–1049. 10.1136/jnnp.2008.157701 19028764 10.1136/jnnp.2008.157701

[13] Nollet JL, Cajander P, Ferris LF, Ramjith J, Omari TI, Savilampi J (2022) Pharyngo-esophageal modulatory swallow responses to bolus volume and viscosity across time. Laryngoscope 132(9):1817–1824. 10.1002/lary.29987 34928519 10.1002/lary.29987 PMC9545908

[14] Omari T (2021) Swallow Gateway™ for High Resolution Pharyngeal and Esophageal Manometry. Flinders Learning Online

[15] Omari T, Ciucci M, Gozdzikowska K, Hernández E, Hutcheson K, Jones C et al (2020) High-resolution pharyngeal manometry and impedance: protocols and metrics-recommendations of a high-resolution pharyngeal manometry international working group. Dysphagia 35(2):281–295. 10.1007/s00455-019-10023-y 31168756 10.1007/s00455-019-10023-y

[16] Omari T, Cock C, Wu P, Szczesniak MM, Schar M, Tack J, Rommel N (2023) Using high resolution manometry impedance to diagnose upper esophageal sphincter and pharyngeal motor disorders. Neurogastroenterol Motil 35(1):e14461. 10.1111/nmo.14461 36121685 10.1111/nmo.14461

[17] Patel B, Legacy J, Hegland KW, Okun MS, Herndon NE (2020) A comprehensive review of the diagnosis and treatment of Parkinson’s disease dysphagia and aspiration. Expert Rev Gastroenterol Hepatol 14(6):411–424. 10.1080/17474124.2020.1769475 32657208 10.1080/17474124.2020.1769475 PMC10405619

[18] Rosenbek JC, Robbins JA, Roecker EB, Coyle JL, Wood JL (1996) A penetration-aspiration scale. Dysphagia 11(2):93–98. 10.1007/bf00417897 8721066 10.1007/BF00417897

[19] Suttrup I, Warnecke T (2016) Dysphagia in Parkinson’s DISEASE. Dysphagia 31(1):24–32. 10.1007/s00455-015-9671-9 26590572 10.1007/s00455-015-9671-9

[20] Suttrup I, Suttrup J, Suntrup-Krueger S, Siemer ML, Bauer J, Hamacher C et al (2017) Esophageal dysfunction in different stages of Parkinson's disease. Neurogastroenterol Motil 29(1). 10.1111/nmo.12915 10.1111/nmo.12915 27477636

[21] Szczesniak MM, Omari TI, Lam TY, Wong M, Mok VCT, Wu JCY et al (2022) Evaluation of oropharyngeal deglutitive pressure dynamics in patients with Parkinson’s disease. Am J Physiol Gastrointest Liver Physiol 322(4):G421–G430. 10.1152/ajpgi.00314.2021 35138164 10.1152/ajpgi.00314.2021

[22] Taira K, Fujiwara K, Fukuhara T, Koyama S, Morisaki T, Takeuchi H (2021) Evaluation of the pharynx and upper esophageal sphincter motility using high-resolution pharyngeal manometry for Parkinson’s disease. Clin Neurol Neurosurg 201:106447. 10.1016/j.clineuro.2020.106447 33421742 10.1016/j.clineuro.2020.106447

