# Supplementary material for: Pharyngeal adaptation to bolus properties in patients with Parkinson’s disease
Source: Eur Arch Otorhinolaryngol. 2024 Jun 12;281(10):5375–83. doi: 10.1007/s00405-024-08774-y (PMC11416356; doi:10.1007/s00405-024-08774-y)
Supplement: Supplementary file 1 — Supplementary file1 (DOCX 32 KB) [file 405_2024_8774_MOESM1_ESM.docx]

**Supplementary Table 1** Number of PD patients presented with abnormal scores for HRIM variables (compared to normative range)

| Measures/ normative data  5^th^ to 95^th^ percentile (unit) | | **IDDSI 0** | | | **IDDSI 2** | | | **IDDSI 4** | | |
| --- | --- | --- | --- | --- | --- | --- | --- | --- | --- | --- |
|  |  | **5ml** | **10ml** | **20ml** | **5ml** | **10ml** | **20ml** | **5ml** | **10ml** | **20ml** |
| PhCI (mmHg.cm.s) | Norms | 109 to 504 | 110 to 504 | 124 to 515 | 105 to 495 | 117 to 492 | 120- 500 | 111 to 498 | 111 to 491 | 122- 520 |
|  | Patients (n) | ↑ 2/16 | ↑ 2/16 | ↑ 3/16 | ↑ 2/16 | ↑ 2/16 | ↑ 2/16 | ↑ 4/16 | ↑ 3/16 | ↑ 2/16 |
| VCI (mmHg.cm.s) | Norms | 17 to 179 | 18 to 211 | 22 to 243 | 17 to 174 | 20 to 204 | 22 to 228 | 19 to 186 | 20 to 205 | 25 to 248 |
|  | Patients (n) | ↑ 1/16 | ↑ 1/16 | ↑ 1/16 | ↑ 1/16 | ↑ 1/16 | ↑ 1/16 | ↑ 1/16 | ↑ 1/16 | ↑ 1/16 |
| MCI(mmHg.cm.s) | Norms | 40 to 233 | 41 to 224 | 44 to 200 | 37 to 243 | 34 to 224 | 42 to 201 | 38 to 240 | 36 to 215 | 40 to 209 |
|  | Patients (n) | ↑ 4/21 | ↑ 2/21 | ↑ 5/21 | ↑ 2/21 | ↑ 2/21 | ↑ 2/21 | ↑ 3/21 | ↑ 2/21 | ↑ 2/21 |
| HPCI (mmHg.cm.s) | Norms | 19 to 159 | 22 to 154 | 23 to 165 | 19 to 170 | 23 to 154 | 24 to 161 | 20 to 162 | 21 to 171 | 25 to 168 |
|  | Patients (n) | ↑ 7/21 | ↑ 6/21 | ↑ 6/21 | ↑ 1/21 | ↑ 3/21 | ↑ 3/21 | ↑ 1/21 | ↑ 2/21 | ↑ 3/21 |
| IBP (mmHg) | Norms | -12 to 17 | -10 to 17 | -8 to 22 | -13 to 15 | -9 to 18 | -5 to 25 | -11 to 17 | -7 to 19 | -4 to 21 |
|  | Patients (n) | ↑ 3/21,↓2/21 | ↑ 4/21,↓1/21 | ↑ 3/21,↓2/21 | None | ↑ 3/21 | ↑ 2/21 | ↑ 1/21,↓1/21 | ↑ 3/21 | ↑ 3/21 |
| UES IRP (mmHg) | Norms | -16 to 2  (-3 to 14) | -15 to 3  (-5 to 20) | -14 to 5 | -14 to 4  (-3 to 25) | -14 to 4  (-3 to 25) | -10 to 7 | -15 to 6 | -12 to 8 | -8 to 13 |
|  | Patients (n) | ↑ 7/21,↓1/21  ↑ 4/21 | ↑ 4/21,↓1/21  ↑ 2/21 | ↑ 4/21,↓1/21 | ↑ 7/21,↓1/21  None | ↑ 5/21,↓1/21  None | ↑ 3/21,↓1/21 | ↑ 4/21 | ↑ 4/21 | ↑ 3/21 |
| UES RT (S) | Norms | 0.4 to 0.7 | 0.4 to 0.7 | 0.4 to 0.8 | 0.4 to 0.7 | 0.4 to 0.7 | 0.4 to 0.8 | 0.4 to 0.7 | 0.4 to 0.7 | 0.4 to 0.7 |
|  | Patients (n) | ↑ 1/21 | ↑2/21, ↓1/21 | ↑ 1/21 | ↑ 1/21 | ↑3/21,↓1/21 | ↑ 2/21 | ↑ 3/21 | ↑ 5/21 | ↑ 1/21 |
| UES MaxAdm (millisiemens-mS) | Norms | 3 to 5 | 3.6 to 6.6 | 4 to 8 | 3.2 to 5.7 | 4.1 to 6.4 | 4.8 to 7.6 | 3.5 to 6.1 | 4.4 to 7.0 | 5.3 to 7.9 |
|  | Patients (n) | ↓ 4/21 | ↓ 3/21 | ↓ 5/21 | ↓ 1/21 | ↓ 6/21 | ↓ 10/21 | ↓ 1/21 | ↓ 4/21 | ↓ 8/21 |
| UES CI (mmHg.cm.s) | Norms | 193 to 1014 | 204 to 1078 | 172 to 1122 | 163 to 979 | 160 to 1097 | 170 to 1119 | 170 to 1118 | 169 to 1060 | 169 to 1240 |
|  | Patients (n) | ↑ 5/21 | ↑ 3/21 | ↑ 4/21 | ↑ 4/21 | ↑ 2/21 | ↑ 2/21 | ↑ 2/21 | ↑ 2/21 | ↑ 2/21 |
| UES BP (mmHg) | Norms | 30- 154 | 28- 166 | 29- 188 | 20 to 151 | 21- 158 | 24- 160 | 22- 136 | 22- 153 | 22- 149 |
|  | Patients (n) | ↑ 3/21 | ↑ 2/21,↓1/21 | ↑ 2/21,↓1/21 | ↑ 2/21,↓1/21 | ↑ 2/21,↓1/21 | ↑ 3/21,↓1/21 | ↑ 2/21 | ↑ 1/21 | ↑ 2/21 |
| UES PeakP | Norms | 112 to 567 | 120 to 593 | 119 to 597 | 118 to 550 | 113 to 539 | 131 to 621 | 123 to 569 | 123 to 575 | 140 to 605 |
|  | Patients (n) | ↑ 3/21 | ↑ 3/21 | ↑ 3/21 | ↑ 4/21 | ↑ 3/21 | ↑ 2/21 | ↑ 3/21 | None | ↑ 2/21 |
| DCL (mS) | Norms | 0.3 to 0.6 | 0.36 to 0.7 | 0.4 to 0.7 | 0.28 to 0.56 | 0.35 to 0.63 | 0.37 to 0.67 | 0.29 to 0.54 | 0.33 to 0.57 | 0.36 to 0.63 |
|  | Patients (n) | None | None | ↑ 2/20 | ↑ 3/21,↓1/21 | ↑ 1/21,↓2/21 | ↑ 3/21,↓1/21 | ↑ 2/21 | ↑ 3/21 | ↑ 3/21 |
| BPT (mS) | Norms | 0.4 to 0.8 | 0.4 to 0.9 | 0.5 to 1.3 | 0.35 to 0.81 | 0.40 to 0.85 | 0.45 to 1.10 | 0.30 to 0.75 | 0.35 to 1.10 | 0.40 to 1.22 |
|  | Patients (n) | ↑ 1/21 | ↑ 4/21,↓1/21 | None | ↑ 4/21 | ↑ 5/21 | ↑ 1/21 | ↑ 2/21 | None | None |
| SRI | Norms | 0 to 4 | 0 to 5 | 0 to 10 | 0.1 to 3.8 | 0.1 to 5.4 | 0.2 to 9.1 | 0.1 to 3.6 | 0.1 to 6.1 | 0.1 to 9.7 |
|  | Patients (n) | ↑ 3/21 | ↑ 2/21 | None | ↑ 2/21 | ↑ 2/21 | None | ↑ 1/21 | ↑ 1/21 | None |

↑ above the 95^th^ percentile of the normative range, ↓below the 5^th^ percentile of the normative range

**Supplementary Table 2** Means and 95% confidence intervals for HRIM metrics

| **Measures** | **IDDSI 0** | | | **IDDSI 2** | | | **IDDSI 4** | | |
| --- | --- | --- | --- | --- | --- | --- | --- | --- | --- |
|  | 5ml  Mean  (95% CI) | 10ml  Mean  (95% CI) | 20ml  Mean  (95% CI) | 5ml  Mean  (95% CI) | 10ml  Mean  (95% CI) | 20ml  Mean  (95% CI) | 5ml  Mean  (95% CI) | 10ml  Mean  (95% CI) | 20ml  Mean  (95% CI) |
| PhCI  (mmHg.cm.s) | 387.23  (299, 475) | 369.64  (279, 459) | 414.81  (317, 512) | 378.71  (292, 464) | 355.65  (285, 455) | 372.56  (292, 452) | 374.26  (281, 466) | 367.82  (272, 463) | 373.95  (302, 445) |
| VCI  (mmHg.cm.s) | 83.28  (58, 107) | 91.36  (53, 129) | 95.78  (58, 133) | 70.88  (51, 90) | 75.73  (52, 98) | 78.67  (54, 102) | 66.30  (46, 86) | 68.84  (44, 93) | 79.06  (53, 105) |
| MCI  (mmHg.cm.s) | 173.57  (124, 222) | 168.44  (114- 222) | 182  (130, 234) | 181.80 (132, 231) | 175.27  (125, 224) | 176.96  (134, 219) | 177.03  (121, 232) | 165.03  (118, 211) | 166.71  (124, 209) |
| HPCI  (mmHg.cm.s) | 130.71  (92, 168) | 109.83  (82, 137) | 136.45  (98, 174) | 126.02  (91, 160) | 119.80  (89, 149) | 118.32  (90, 146) | 131.43  (87, 175) | 134.68  (81, 188) | 128.17  (93, 163) |
| UES IRP  (mmHg) | -0.42  (-5.84, 4.99) | -1.70  (-6.3, 2.9) | -0.45  (-5.4, 4.5) | -3.77  (-7.86, 0.32) | -1.92  (-5.39, 1.55) | -0.99  (-4.47, 2.49) | -2.55  (-5.85, 0.74) | -0.03  (-4.22, 4.14) | 1.53  (-3.31, 6.38) |
| UES RT  (s) | 0.57  (0.51, 0.63) | 0.61  (0.54, 0.67) | 0.65  (0.60, 0.70) | 0.59  (0.53, 0.65) | 0.61  (0.56, 0.66) | 0.64  (0.58, 0.70) | 0.58  (0.52, 0.63) | 0.59  (0.54, 0.65) | 0.67  (0.58, 0.75) |
| UES Max Adm (mS) | 3.32  (3.10, 3.53) | 3.99  (3.7, 4.2) | 4.42  (4.0, 4.8) | 3.78  (3.48, 4.08) | 4.50  (4.21, 4.79) | 4.92  (4.59, 5.25) | 4.20  (3.93, 4.47) | 4.76  (4.47, 5.05) | 5.28  (4.96, 5.59) |
| IBP  (mmHg) | 6.83  (-1.93, 5.61) | 10.6  (2.23, 19.01) | 8.79  (1.31, 16.27) | 3.39  (-3.58, 0.37) | 7.81  (1.07, 14.54) | 9.53  (2.56, 16.50) | 2.91  (-2.0, 7.83) | 6.16  (-0.52, 2.84) | 8.48  (1.79, 15.17) |
| UES CI (mmHg.cm.s) | 772.76  (575, 969) | 740.29  (492, 987) | 852.31  (600, 1104) | 712.79  (448, 976) | 675.61  (452, 898) | 716.86  (508, 925) | 661.24  (477, 845) | 631.72  (454, 809) | 747.82  (511, 984) |
| UES BP  (mmHg) | 120.81  (82-158) | 110.23  (66, 153) | 118.27  (76, 160) | 108.21  (71, 144) | 96.04  (65, 127) | 106.23  (74, 137) | 104.05  (70, 137) | 95.04  (67, 122) | 96.53  (61, 43) |
| UES PeakP  (mmHg) | 397.98  (326, 469) | 384.18  (300, 467) | 422.03  (343, 500) | 370.32  (283, 457) | 368.25  (297, 438) | 409.69  (323, 496) | 379.83  (296, 463) | 365.03  (288, 441) | 408.83  (319, 498) |
| DCL  (mS) | 0.51  (0.46, 0.55) | 0.55  (0.49, 0.60) | 0.59  (0.53, 0.64) | 0.48  (0.41, 0.54) | 0.54  (0.48, 0.59) | 0.54  (0.47, 0.60) | 0.43  (0.38, 0.49) | 0.48  (0.43, 0.54) | 0.55  (0.48, 0.61) |
| BPT  (mS) | 0.62  (0.52, 0.72) | 0.67  (0.54, 0.79) | 0.74  (0.61, 0.86) | 0.60  (0.50, 0.70) | 0.66  (0.52, 0.79) | 0.70  (0.58, 0.82) | 0.54  (0.46, 0.61) | 0.58  (0.51, 0.64) | 0.71  (0.62, 0.80) |
| SRI | 2.09  (0.45, 3.73) | 2.01  (0.76, 3.27) | 1.83  (0.68, 2.97) | 1.09  (0.05, 2.13) | 1.51  (0.42, 2.60) | 2.16  (0.63, 3.68) | 1.09  (0.42, 1.77) | 1.60  (0.33, 2.86) | 1.93  (0.48, 3.37) |

Model estimated marginal means and 95% confidence intervals are presented. CI: Confidence interval, UES- Upper-esophageal sphincter, PCI- Pharyngeal contractile integral, VCI-Velopharyngeal contractile integral, MCI- Mesopharyngeal contractile integral, HPCI- Hypopharyngeal contractile integral, PeakP- Peak Pressure, UES IRP- UES integrated relaxation pressure, UES RT-UES relaxation time, UESMaxAdm- UES maximum admittance, IBP- Intra-bolus distension pressure, UES CI- UES contractile integral, UES BP- UES basal pressure, UES PeakP- UES peak pressure, DCL- Pharyngeal Distension-Contraction Latency, BPT- Bolus Presence Time, SRI- Swallow risk index.

Supplementary Table 3 Swallow variables' main effects in relation to severity scores

| Bolus condition | | PDQ-8 group effects | EAT-10 group effects |
| --- | --- | --- | --- |
|  |  | (S.score < 33), n= 11,  (S. score ≥ 33), n= 10 | (Raw score < 7), n= 10  (Raw score ≥ 7), n= 11 |
| IDDSI 0  (Thin liquid) | 5ml | None | UES BP  *(Z =* - 2.04, *p =* 0.04) UES MaxAdm  (*Z =* - 2.53, *p =* 0.01) |
|  | 10ml | None | UES BP  *(Z =* - 1.97, *p =* 0.04) |
|  | 20ml | None | None |
| IDDSI 2  (Mildly thick liquid) | 5ml | None | UES MaxAdm  *(Z =* - 3.09, *p =* 0.002) |
|  | 10ml | None | UES MaxAdm  *(Z =* - 2.60, *p =* 0.009) |
|  | 20ml | None | UES MaxAdm  *(Z =* - 2.60, *p =* 0.009) |
| IDDSI 4  (Extremely thick liquid) | 5ml | None | VCI  *(t =* 2.71, *p =* 0.01) |
|  | 10ml | None | VCI  *(t =* 2.69, *p =* 0.01) UES MaxAdm (*Z =* - 1.97, *p =* 0.04) |
|  | 20ml | None | None |

S.Score: Standardized score, None: no significant effects on any of the HRIM measures
